# Helmholtz’s decomposition for compressible flows and its application to computational aeroacoustics

**DOI:** 10.1007/s42985-020-00044-w

**Published:** 2020-11-06

**Authors:** Stefan Schoder, Klaus Roppert, Manfred Kaltenbacher

**Affiliations:** 1 TU Graz, Institute of Fundamentals and Theory in Electrical Engineering, Inffeldgasse 18, 8010 Graz, Austria; 2 TU Wien, Institute of Mechanics and Mechatronics, Getreidemarkt 9, 1060 Wien, Vienna Austria

**Keywords:** Helmholtz’s decomposition, Compressible flows, Aeroacoustic, FEM, 76-10, 68-04

## Abstract

The Helmholtz decomposition, a fundamental theorem in vector analysis, separates a given vector field into an irrotational (longitudinal, compressible) and a solenoidal (transverse, vortical) part. The main challenge of this decomposition is the restricted and finite flow domain without vanishing flow velocity at the boundaries. To achieve a unique and $L_2$-orthogonal decomposition, we enforce the correct boundary conditions and provide its physical interpretation. Based on this formulation for bounded domains, the flow velocity is decomposed. Combining the results with Goldstein’s aeroacoustic theory, we model the non-radiating base flow by the transverse part. Thereby, this approach allows a precise physical definition of the acoustic source terms for computational aeroacoustics via the non-radiating base flow. In a final simulation example, Helmholtz’s decomposition of compressible flow data using the finite element method is applied to an overflowed rectangular cavity at Mach 0.8. The results show a reasonable agreement with the source data and illustrate the distinct parts of the Helmholtz decomposition.

## Introduction

Based on the methods used in computational aeroacoustics, we combine the presented Helmholtz decomposition with Goldstein’s aeroacoustic theory [11].Since early computational aeroacoustics (CAA), numerical methodologies have been proposed. Every method tries to overcome the challenges of effective and accurate computation of the radiated sound. Two important difficulties, which have to be considered for the simulation of flow sound problems, are summarized as [1, 2, 4]:

1. *Energy disparity and acoustic inefficiency:* A large disparity between the flow energy and the radiated acoustic energy of flow-induced sound has been observed. For example, the total radiated power of a turbulent jet scales with $O(\mathcal {U}^8 / c^5)$ ($\mathcal {U}$ is the characteristic flow velocity and *c* the speed of sound), whereas the power of the turbulent jet scales with $O(\mathcal {U}^3)$. This implies that direct simulation of flow and sound must be able to resolve very different scales and amplitudes. For low Mach number applications, one solution is to use a hybrid aeroacoustic workflow [4].
2. *Length scale disparity:* In low Mach number aeroacoustic applications, a large disparity between the size of a turbulent eddy and the wavelength of the generated acoustic sound exists. The turbulent eddy has a characteristic length scale of $l_\mathrm{v}$, velocity $\mathcal {U}$, a eddy lifetime $l_\mathrm{v}/\mathcal {U}$ and a frequency *f*, and radiates acoustic waves with the same characteristic frequency but with a larger length scale
  1 $$\begin{aligned} \lambda \propto \frac{l_\mathrm{v}}{\text {Ma}}, \end{aligned}$$
  than the size of the turbulent eddy. In (1) $\text {Ma} = \mathcal {U}/c$ denotes the Mach number. This implies that an acoustics simulation has different requirements for the discretization and for the relevant domain size, to fit several wavelength. For low Mach number applications, one efficient simulation solution is to decouple flow and acoustics by a hybrid workflow [4].

As a consequence of these two challenges at low Mach number, flow and acoustics are often simulated separately.Modeling flow-induced acoustics, the partial differential equation has a hyperbolic left-hand side (d’Alembert operator or wave operator $\Box$) and a generic right-hand side $\mathbf{RHS} (\star )$

2 $$\begin{aligned} \Box p^\prime = \mathbf{RHS} ( p, \mathbf {u}, \rho , ... )\,. \end{aligned}$$

Lighthill’s inhomogeneous wave equation perfectly fits into this class of equations [5]. Thereby, Lighthill’s $\mathbf{RHS} (\star )$ contains not only source terms but also interaction terms between the sound and flow field, which includes effects, such as convection and refraction of the sound by the flow. Regarding physics, the full set of compressible flow equations (including acoustics) has to be solved to calculate the right-hand side. The flow simulation must already resolve the acoustics inside $\mathbf{RHS} (\star )$, which is a challenge because computational errors itself may strongly disturb the physical sound radiation [6, 7]. Phillips and Lilley [8, 9] moved interaction effects, at least to some extent, to the wave operator $\Box$ and predicted certain aspects of jet-noise accurately. Lighthill’s wave operator neglects these effects, and often, Lighthill’s source term does not include these effects due to the restricted numerical resolution of the precomputed flow simulation [10].

In the year 2003, Goldstein [11] proposed a method to split flow variables $(p, \mathbf {u}, ...)$ into a base flow $\tilde{\star }$ and a remaining component $\star ^\prime$

3 $$\begin{aligned} \star = \tilde{\star }+ \star ^\prime \,. \end{aligned}$$

Although Goldstein’s idea has a different perspective, the separation shows distinctive features of sound in the source terms. In this contribution, we develop a theory to derive aeroacoustic wave equations. Therefore, we define a non-radiating flow field as a flow that contains no sound but has the potential to generate sound. Examples for a non-radiating base flow are: an incompressible flow in combination with the expansion about the incompressible flow (EIF) [3], a Reynolds averaged Navier-Stokes (RANS) solution in combination with synthetic noise generation and radiation (SNGR), or simply a steady background flow. The remaining component is the perturbation upon the base flow and describes all radiating components, including the sound field.

Applying the decomposition to the right-hand side of the wave equation yields

4 $$\begin{aligned} \Box p^\prime = \mathbf{RHS} ( \tilde{p}, \tilde{\mathbf {u}}, \tilde{\rho }, p^\prime , \mathbf {u}^\prime , \rho ^\prime , ... )\,. \end{aligned}$$

Interaction terms can be moved to the differential operator to take, e.g., convection and refraction effects or even nonlinear interactions into account. This approach was applied in the theories of Phillips and Lilley, and the derivation of perturbation equations [12–14]. Following this modeling approach, the main interaction effects between the flow and the acoustic field are modeled by a proper wave operator $\tilde{\Box }$ and the right-hand side, which corresponds the physical sound sources, contains only the base flow

5 $$\begin{aligned} \tilde{\Box }p^\prime = \mathbf{RHS} ( \tilde{p}, \tilde{\mathbf {u}}, \tilde{\rho }, ...)\,. \end{aligned}$$

Based on this theory, an essential building block for the construction of a non-radiating base flow is the Helmholtz decomposition. Within this contribution, we introduce Helmholtz’s decomposition to separate a velocity field into vortical and compressible flow structures. The origin of such a decomposition reaches far into the history of science [15, 16]. Many disciplines have used this method, like electrodynamics [17, 18], fluid dynamics [19] and computer visualization [21].

**Fig. 1:**
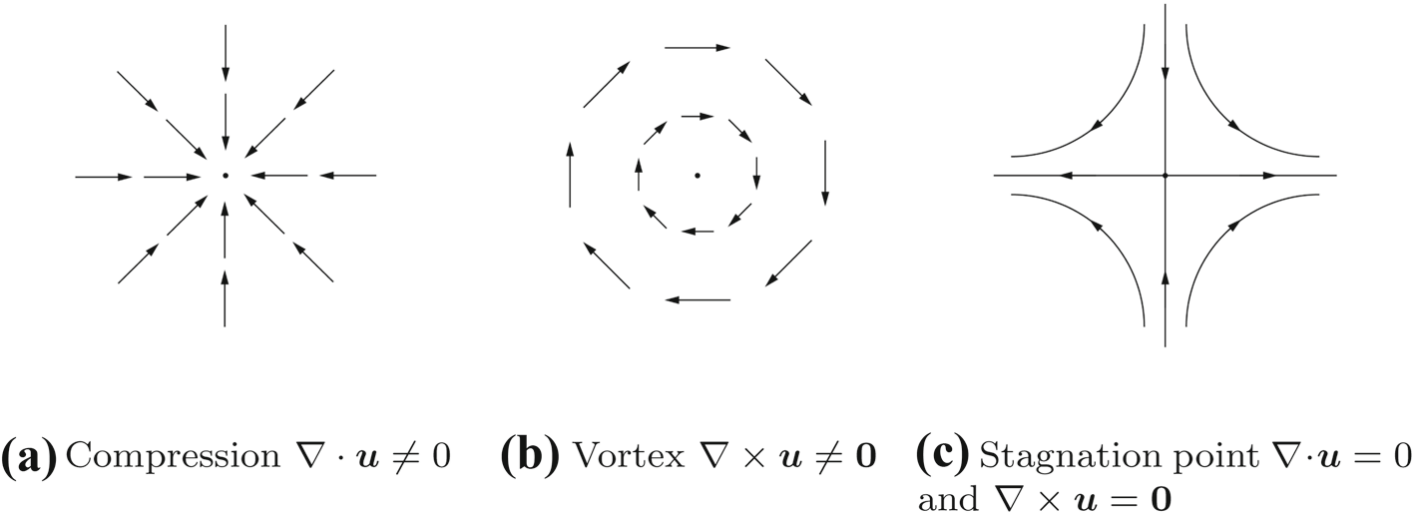
Three different mode shapes illustrated in terms of the flow velocity. **a** Compression of a fluid field. **b** Vortex inside a fluid. **c** Stagnation point of a potential flow (streamlines)

The decomposition isolates the irrotational part (longitudinal process - compressible) from the solenoidal part (transverse process - vortical). This separation allows for studying vortex dynamics using potential representation and stream functions. Based on fluid dynamics, the motion of a continuum point is a combination of three independent shapes [19] (see Fig. 1).

Firstly, an isotropic expansion proportional to the volumetric rate of expansion $\nabla \cdot {\varvec{u}} = \nabla \cdot {\varvec{u}}_\mathrm{c}$ (see Fig. 1a). This field component ${\varvec{u}}_\mathrm{c}$ is described by a scalar potential $\phi$ associated with the compressibility of the fluid. The scalar velocity potential can be computed by solving the Poisson equation

6 $$\begin{aligned} \nabla \cdot \nabla \phi = \nabla \cdot {\varvec{u}} \, . \end{aligned}$$

Secondly, a rigid-body rotation at an angular velocity of $\frac{1}{2} \nabla \times {\varvec{u}} = \frac{1}{2}\nabla \times {\varvec{u}}_\mathrm{v}$ (see Fig. 1b. The vorticity and its dynamics describe the vortical and incompressible flow structures, which may be described by a vector potential ${\varvec{A}}$. The vector velocity potential can be computed by solving the curl-curl equation

7 $$\begin{aligned} \nabla \times \nabla \times {\varvec{A}} = \nabla \times {\varvec{u}} \, . \end{aligned}$$

Thirdly, irrotational deformation without volume change (see Fig.  1(c). The theory of potential flow equations describes this velocity component, which is both divergence-free $\nabla \cdot {\varvec{u}}_\mathrm{h} = 0$ and curl-free $\nabla \times {\varvec{u}}_\mathrm{h} = {\varvec{0}}$ [19]. This field is called the harmonic component of the Helmholtz decomposition. A potential flow has therefore an irrotational solenoidal vector field ${\varvec{u}}_\mathrm{h} = \nabla \Phi = \nabla \times {\varvec{B}}$ that is computed by the Laplace equation of the velocity potential $\Phi$

8 $$\begin{aligned} \nabla \cdot \nabla \Phi = 0 \, \end{aligned}$$

or the homogeneous curl-curl equation of the general stream function ${\varvec{B}}$

9 $$\begin{aligned} \nabla \times \nabla \times {\varvec{B}} = {\varvec{0}} \, , \end{aligned}$$

with respect to the flow boundary condition at the inlet and outlet of the flow domain. As a consequence, the potential flow is the homogeneous form of the partial differential Eqs. (6) and (7), respectively. Due to the boundary condition, a potential flow (harmonic part) will be part of the solution of the decomposed vector fields. The herein developed boundary condition is defining the imposed harmonic part in advance.

Based on Helmholtz’s decomposition [19, 28–30], new formulations of the equations [22, 23], simulation solver [24–27], and boundary conditions [15] are developed. Regarding aeroacoustic, De Roeck and Desmet [27] assumed a convex domain when computing Helmholtz’s decomposition. The generalization to non-convex domains involves additional treatment [51]. As non-convex domains occur in real-world applications (e.g. fans, cavities, and duct systems), Schoder *et al.* [31, 35] address them by the curl-curl operator in combination with $H(\mathrm {curl},\Omega )$ conforming finite elements. In contrast to previous work, we show the finite element method to compute Helmholtz’s decomposition with the appropriate function space for the vector potential $H(\mathrm {curl},\Omega )$. To compute the vector potential, we have two systems of equations at hand that converge in the limit of small regularization parameters. Additionally, the relation to potential flow theory is discussed in detail. This is especially important since the method is dealing with finite domains. Furthermore, within the contribution, the results are compared by a quantitative measure systematically.

The rest of this paper is structured as follows. Section  2 introduces Helm-holtz’s decomposition and its extensions to finite domains. Section 3 derives the weak formulation based on the curl-curl equation, which is discretized by edge elements. Besides, we verify the developed boundary conditions by computations of a cylinder in crossflow. In Section  4, we apply the method to a compressible flow field over a rectangular cavity at Mach 0.8 [31], followed by a summarizing conclusion.

## The Helmholtz decomposition for finite domains

Helmholtz’s decomposition is a fundamental theorem in vector analysis and frequently applied fluid dynamics [18, 28–30, 32], to obtain from a given flow field its distinct parts. After separating the irrotational from the solenoidal part of the flow field ${\varvec{u}}$, the potential representation is used to study vortex dynamics and compression isolated from each other.

### Theorem 1

*Every vector field* ${\varvec{u}}$, $\mathcal {C}^1$ *smooth, on a simply connected domain* $\Omega \subset \mathbb {R}^3$ *with the property* $\lim _{r \rightarrow \infty } {\varvec{u}}(r) = {\varvec{0}}$ *of a radial coordinate* $r = ||{\varvec{x}}||_2$ *with* ${\varvec{x}} \in \Omega$ , *can be decomposed in L*$_2$-*orthogonal velocity field components*

10 $$\begin{aligned} {\varvec{u}} = {\varvec{u}}_\mathrm{v} + {\varvec{u}}_\mathrm{c} = \nabla \times {\varvec{A}}_\mathrm{v} + \nabla \phi _\mathrm{c} \, , \end{aligned}$$

with the vector potential ${\varvec{A}}_\mathrm{v}$ satisfying the condition $\nabla \cdot {\varvec{A}}_\mathrm{v} = 0$ additionally, the scalar potential $\phi _\mathrm{c}$, a vortical velocity component ${\varvec{u}}_\mathrm{v}$ satisfying $\nabla \cdot {\varvec{u}}_\mathrm{v} = 0$, and a compressible velocity component ${\varvec{u}}_\mathrm{c}$ satisfying $\nabla \times {\varvec{u}}_\mathrm{c} = {\varvec{0}}$ (Proof can be found in [28]).

**Fig. 2:**
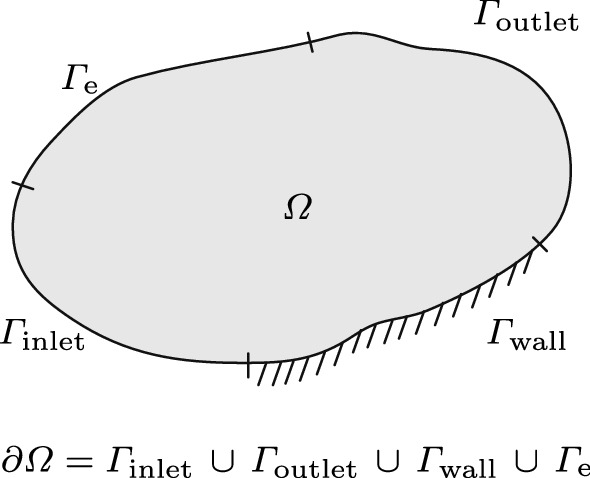
Schematic and definition of the decomposition domain $\Omega$, the boundary of the domain $\partial \Omega$ that consists of the boundary sections: wall $\Gamma _\mathrm{wall}$, inlet $\Gamma _\mathrm{inlet}$, outlet $\Gamma _\mathrm{outlet}$, and essential boundary $\Gamma _\mathrm{e}$

Because, for numerical simulations, the velocity field ${\varvec{u}}$ is just known on a finite domain (see Fig. 2), we cannot apply 1. For vanishing flow fields inside the simulation domain 1 is satisfied for simply connected domains [33]. This means that the velocity field is concentrated inside the domain and is already zero ${\varvec{u}} = {\varvec{0}}$ at the domain boundaries, which allows a virtual prolongation of the velocity field towards infinity and therefore the prolongated flow field satisfies the assumption $\lim _{r \rightarrow \infty } {\varvec{u}}(r) = {\varvec{0}}$.

Without vanishing fields at the boundaries ${\varvec{u}} \ne 0$ on $\partial \Omega$ (no virtual prolongation of the velocity field towards infinity is possible), Helmholtz’s decomposition can cause a non-unique and a non-$L_2$-orthogonal decomposition [34]. Physically, this leads to a non-unique separation of the harmonic field (potential flow part) due to the boundary values. On a general finite domain, 1 reads as follows.

### Theorem 2

*Every square integrable vector field* ${\varvec{u}} \in [L_2(\Omega )]^3$ ($\mathcal {C}^1$ *smooth*) *on a Lipschitz domain* $\Omega \subseteq \mathbb {R}^3$, *has an L*$_2$-*orthogonal decomposition*

11 $$\begin{aligned} {\varvec{u}} = {\varvec{u}}_\mathrm{v} + {\varvec{u}}_\mathrm{c} + {\varvec{u}}_\mathrm{h} = \nabla \times {\varvec{A}}_\mathrm{v} + \nabla \phi _\mathrm{c} + {\varvec{u}}_\mathrm{h} \, , \end{aligned}$$

with the vector potential ${\varvec{A}}_\mathrm{v} \in H(\mathrm {curl},\Omega )$, the scalar potential $\phi _\mathrm{c} \in H^1(\Omega )$ and the harmonic component ${\varvec{u}}_\mathrm{h} \in [L_2(\Omega )]^3$.The vortical velocity component ${\varvec{u}}_\mathrm{v}$ satisfies $\nabla \cdot {\varvec{u}}_\mathrm{v} = 0$, and a compressible velocity component ${\varvec{u}}_\mathrm{c}$ satisfies $\nabla \times {\varvec{u}}_\mathrm{c} = {\varvec{0}}$, and a harmonic component ${\varvec{u}}_\mathrm{h}$ satisfies both $\nabla \cdot {\varvec{u}}_\mathrm{h} = 0$ and $\nabla \times {\varvec{u}}_\mathrm{h} = {\varvec{0}}$. (Proof can be found in [19, 36])

If the decomposition is either computed in the scalar-potential formulation (solve the governing equation for $\phi _\mathrm{c}$) or in the vector potential formulation (solve the governing equation for ${\varvec{A}}_\mathrm{v}$). Depending on what equation is solved, the obtained components include the harmonic component (homogeneous solution of the solved partial differential equation) due to the boundary values of the solved partial differential equation. Therefore, we mark both potentials with a star superscript.

12 $$\begin{aligned} {\varvec{u}}= {} {\varvec{u}}_\mathrm{v} + {\varvec{u}}_\mathrm{c} + {\varvec{u}}_\mathrm{h} = \nabla \times {\varvec{A}}^*_\mathrm{v} + \nabla \phi ^*_\mathrm{c} = {\varvec{u}}^*_\mathrm{v} + {\varvec{u}}^*_\mathrm{c} \end{aligned}$$

13 $$\begin{aligned} {\varvec{u}}^*_\mathrm{c}= {} {\varvec{u}}_\mathrm{c} + \alpha {\varvec{u}}_\mathrm{h} \end{aligned}$$

14 $$\begin{aligned} {\varvec{u}}^*_\mathrm{v}= {} {\varvec{u}}_\mathrm{v} + \beta {\varvec{u}}_\mathrm{h} \end{aligned}$$

15 $$\begin{aligned} \alpha + \beta= & {} 1 \end{aligned}$$

For any decomposition that is possible, we like to include the harmonic part into the compressible ${\varvec{u}}^*_\mathrm{c}$ or the vortical part ${\varvec{u}}^*_\mathrm{v}$ by choosing appropriate boundary conditions. The $L_2$-orthogonality of the decomposed components of 2 requires

16 $$\begin{aligned} ({\varvec{u}}^*_\mathrm{v},{\varvec{u}}^*_\mathrm{c}) = \int _\Omega {\varvec{u}}^*_\mathrm{v} \cdot {\varvec{u}}^*_\mathrm{c} \mathrm {d} x = 0 \, . \end{aligned}$$

Inserting the definition of the joint velocities ${\varvec{u}}^*_\mathrm{v}$ and ${\varvec{u}}^*_\mathrm{c}$) as a consequence of the joint potentials (${\varvec{A}}^*_\mathrm{v}$ and $\phi ^*_\mathrm{c}$) and applying the $L_2$-orthogonality of the two components yields

17 $$\begin{aligned} ({\varvec{u}}^*_\mathrm{v},{\varvec{u}}^*_\mathrm{c}) = \int _\Omega {\varvec{u}}^*_\mathrm{v} \cdot {\varvec{u}}^*_\mathrm{c} \mathrm {d} x = \int _\Omega \alpha \beta {\varvec{u}}_\mathrm{h} \cdot {\varvec{u}}_\mathrm{h} \mathrm {d} x = 0 \, , \end{aligned}$$

and therefore a condition for $\alpha$ and $\beta$ to be satisfied. In order to be orthogonal, the harmonic part ${\varvec{u}}_\mathrm{h}$ must be either included into the compressible component ${\varvec{u}}^*_\mathrm{c}$ ($\alpha =1$ and $\beta =0$) or into the vortical component ${\varvec{u}}^*_\mathrm{v}$ ($\alpha =0$ and $\beta =1$). We prefer $\alpha =0$ and $\beta =1$, since for low Mach numbers the obtained vortical field recovers the incompressible flow solution. Using the $L_2$-orthogonality condition, we derive a unique boundary condition that leads to a distinct union of the harmonic field (which represents the exterior influence of the decomposed vector field ${\varvec{u}}$) into the vortical part with $\alpha =0$ and $\beta =1$.

The partial differential equation for the scalar potential $\phi ^*_\mathrm{c} \in H^1(\Omega )$ (also known as scalar-potential formulation or Poisson problem) is associated with the compressible part and the property $\nabla \times {\varvec{u}}^*_\mathrm{c} = 0$. According to the choice of $\alpha$ and $\beta$, Helmholtz’s decomposition reads then as

18 $$\begin{aligned} {\varvec{u}}= & {} \nabla \times {\varvec{A}}^*_\mathrm{v} + \nabla \phi ^*_\mathrm{c} \, , \end{aligned}$$

19 $$\begin{aligned} \nabla \phi ^*_\mathrm{c}= & {} \nabla \phi _\mathrm{c} + \nabla \phi _\mathrm{h} = {\varvec{u}}^*_\mathrm{c} = {\varvec{u}}_\mathrm{c} + \alpha {\varvec{u}}_\mathrm{h}\vert _{\alpha =0} = {\varvec{u}}_\mathrm{c}\, . \end{aligned}$$

By taking the divergence of (18), we obtain a scalar valued Poisson equation with the dilatation $\nabla \cdot {\varvec{u}}$ as forcing

20 $$\begin{aligned} \nabla \cdot \nabla \phi ^*_\mathrm{c} = \nabla \cdot {\varvec{u}}\, . \end{aligned}$$

This scalar Poisson Eq. (20) can be solved computationally efficient and may even be calculated inside the flow solve. Please note that (20) has a similar structure as the pressure correction equation that is solved by flow solvers (e.g. [46]). However, (20) suffers from two main drawbacks:

- The computational domain of the scalar potential should capture compressible effects e.g. acoustics. Acoustic radiation, in a free field configuration, reaches far into the surrounding since the acoustic field only decays by $O(1/||{\varvec{x}}||_2)$. This slow decay results either in a large domain or in an involved boundary condition that accurately fulfills free field behavior. A possible solution strategy for elliptic differential equations is the infinite mapping layer [37].
- The second issue arises for non-convex domains with a $C^0$ smooth boundary, like reentrant corners [31, 35]. For such flow domains, the computation of the scalar potential leads to a singular point at a reentrant corner and corrupts the solution. Using FEM, a graded mesh can treat this singularity. However, in most cases, these reentrant corners are locations where aeroacoustic sources are present and radiate intensely [47]. Therefore, these regions have to be treated carefully.

Caused by these two drawbacks, the application of the computationally efficient scalar-potential formulation is limited to simply connected convex domains. We suggest the application of the vector potential formulation.

The Helmholtz decomposition, formulated by the vector potential ${\varvec{A}}^{*}_\mathrm{v} \in H(\mathrm {curl},\Omega )$ (also known as vector potential formulation or curl-curl problem), is associated with the incompressible part and the property $\nabla \cdot {\varvec{u}}^{*}_\mathrm{v} = 0$. We aim to decompose the velocity field by

21 $$\begin{aligned} {\varvec{u}}= {} \nabla \times {\varvec{A}}^*_\mathrm{v} + \nabla \phi ^*_\mathrm{c} \, , \end{aligned}$$

22 $$\begin{aligned} \nabla \times {\varvec{A}}^*_\mathrm{v} = {\varvec{u}}^*_\mathrm{v}= {} {\varvec{u}}_\mathrm{v} + \beta {\varvec{u}}_\mathrm{h}\vert _{\beta =1} = {\varvec{u}}_\mathrm{v} + {\varvec{u}}_\mathrm{h} \end{aligned}$$

such that the harmonic part is united with the vortical part ($\alpha =0$ and $\beta =1$) defined by the joint vector potential ${\varvec{A}}^*_\mathrm{v}$. In the case of holes inside the domain, these holes enrich the function space. For example, imagine a flow around a cylinder at very low Mach number and then decompose this flow. As a result the vortical part of the flow simulation will converge to the incompressible flow solution as the Mach number approaches zero [14] (see Sect. 3.2). By taking the curl of equation (21), the curl-curl equation for the vector potential ${\varvec{A}}^*_\mathrm{v}$ is obtained

23 $$\begin{aligned} \nabla \times \nabla \times {\varvec{A}}^*_\mathrm{v} = \nabla \times {\varvec{u}} = {\varvec{\omega }}\, , \end{aligned}$$

forced by the vorticity $\varvec{\omega }= \nabla \times {\varvec{u}}$, with $\nabla \cdot \varvec{\omega }= \nabla \cdot \nabla \times {\varvec{u}} = 0$. The function space must also ensure the orthogonality of the decomposed components. Applying the integration by parts to the $L_2$-orthogonality leads to

24 $$\begin{aligned} ({\varvec{u}}^*_\mathrm{v},{\varvec{u}}^*_\mathrm{c})= (\nabla \times {\varvec{A}}^*_\mathrm{v},{\varvec{u}}^*_\mathrm{c}) = ({\varvec{A}}^*_\mathrm{v},\underbrace{\nabla \times {\varvec{u}}^*_\mathrm{c}}_{=0}) + \int _{\partial \Omega } {\varvec{A}}^*_\mathrm{v} \cdot (\underbrace{{\varvec{u}}^*_\mathrm{c}}_{{\varvec{u}} - \nabla \times {\varvec{A}}^*_\mathrm{v}} \times {\varvec{n}} ) \mathrm {d}s \nonumber \\= \int _{\partial \Omega } {\varvec{A}}^*_\mathrm{v} \cdot ({\varvec{u}} - \nabla \times {\varvec{A}}^*_\mathrm{v}) \times {\varvec{n}} \mathrm {d} s = 0 \, , \end{aligned}$$

as uniqueness condition at the boundary $\partial \Omega$. We ensure that the harmonic part is united with the vortical part by assuming $({\varvec{u}} - \nabla \times {\varvec{A}}^*_\mathrm{v}) \times {\varvec{n}} ={\varvec{0}}$. Based on the orthogonality condition (24), typical flow boundaries in combination with the curl-curl problem can be identified.

*Wall* For a rigid and perfectly smooth wall, the compressible velocity component in tangential direction ${\varvec{u}}_\mathrm{c} \times {\varvec{n}} = {\varvec{0}}$ is zero. A no slip and non-penetrating wall boundary requires that the overall tangential flow velocity is equal to the wall movement ${\varvec{u}}_\mathrm {wall}$ in tangential direction

25 $$\begin{aligned} {\varvec{u}}^*_\mathrm{v} \times {\varvec{n}} = (\nabla \times {\varvec{A}}^*_\mathrm{v}) \times {\varvec{n}}= {\varvec{u}}_\mathrm {wall} \times {\varvec{n}} \, . \end{aligned}$$

Stationary, rigid walls, with a no-slip condition ${\varvec{u}}_\mathrm {wall} = {{0}}$, enforce a homogeneous Neumann boundary for the vector potential ($\nabla \times {\varvec{A}}^*_\mathrm{v}) \times {\varvec{n}}={{0}}$).

*Inlet and Outlet* At the inlet and the outlet the tangential velocity is described by

26 $$\begin{aligned} {\varvec{u}}^*_\mathrm{v} \times {\varvec{n}}= (\nabla \times {\varvec{A}}^*_\mathrm{v}) \times {\varvec{n}}= {\varvec{u}}_\mathrm {inlet/outlet} \times {\varvec{n}} \,. \end{aligned}$$

Here, we assume that the tangential velocity component of the total field is dominated by the vortical part ${\varvec{u}}^*_\mathrm{v}$ to a sufficient extent (${\varvec{u}}^*_\mathrm{c} \times {\varvec{n}} \vert _{\partial \Omega } \ll {\varvec{u}} \times {\varvec{n}} \vert _{\partial \Omega }$), which leads to ${\varvec{u}}^*_\mathrm{v} \times {\varvec{n}} \vert _{\partial \Omega } := {\varvec{u}} \times {\varvec{n}}\vert _{\partial \Omega }$. Three facts support this approximation. Firstly, the amplitudes of the acoustic perturbation are small. Additionally, if the numerical setup for computing the compressible flow dissipates wave (sponge zone), the waves do not travel until the free boundaries. Secondly, the acoustic perturbations are a longitudinal process. Therefore sponge zones are arranged mostly orthogonal to the radiation direction. So, the compressible part in the tangential velocity component is weighted by the sine of the relative angle, which further reduces the amplitudes. Thirdly, if the numerical setup uses simple absorbing boundary conditions, the prescribed radiation condition at flow boundaries is optimal for normal wave impingement. Consequently, the domains are designed to satisfy normal wave impingement. However, for high Mach number flows, this assumption will not hold.

Based on the definition of the inhomogeneous Neumann condition, the weak formulation of (23) reads as follows: Find ${\varvec{A}} \in V$ such that

27 $$\begin{aligned} \int _{\Omega } (\nabla \times {\varvec{A}}') \cdot (\nabla \times {\varvec{A}}) \mathrm {d}x - \int _{\partial \Omega } {\varvec{A}}' \cdot ({\varvec{u}}^{*}_\mathrm{v} \times {\varvec{n}}) \mathrm {d}s = \int _{\Omega } {\varvec{A}}' \cdot {\varvec{\omega }}\mathrm {d} x \end{aligned}$$

is fulfilled for all ${\varvec{A}}' \in W$ with

$$\begin{aligned} V:= &\, {} H_{{\varvec{g}}_\mathrm{e}}(\mathrm{curl},\, \Omega ) = \{ {\varvec{B}} \in [L_2]^3 | \ \nabla \times {\varvec{B}} \in [L_2]^3,\, {\varvec{B}}(x) \times {\varvec{n}} = {\varvec{g}}_\mathrm{e}(x) \, \mathrm {on} \, \Gamma _e \} \\ W:= &\, {} H_0(\mathrm{curl},\, \Omega ) = \{ {\varvec{C}} \in [L_2]^3 | \ \nabla \times {\varvec{C}} \in [L_2]^3,\, {\varvec{C}}(x) \times {\varvec{n}} = {{0}} \, \mathrm {on} \, \Gamma _e \} \, , \end{aligned}$$

with ${\varvec{g}}_\mathrm{e}(x)$ being the essential boundary values on $\Gamma _e$. Although, the vector potential is in the desired function space, the vector potential has to be divergence-free (see 2). Since, the vector potential is only unique up to gradient fields $\Pi$

28 $$\begin{aligned} {\varvec{A}}^+ = {\varvec{A}}^* + \nabla \Pi \, . \end{aligned}$$

When the incompressible velocity is computed, the gradient field vanishes

29 $$\begin{aligned} {\varvec{u}}^{*}_\mathrm{v} = \nabla \times ({\varvec{A}}^{*}_\mathrm{v} - \nabla \Pi ) = \nabla \times {\varvec{A}}^{*}_\mathrm{v} \, . \end{aligned}$$

In order to select a unique solution, the vector potential is gauged. The Coulomb gauge condition

30 $$\begin{aligned} \nabla \cdot {\varvec{A}}^{*}_\mathrm{v}=0 \end{aligned}$$

may be incorporated by a Lagrange constraint or mass regularization. For simplicity, the boundary forcing of the weak formulation is neglected. Introducing a Lagrange constraint, we will solve a saddle point problem for ${\varvec{A}} \in H_0(\mathrm {curl},\Omega )$ and $\varphi \in H_0^1(\Omega )$

31 $$\begin{aligned} \int _{\Omega } (\nabla \times {\varvec{A}}') \cdot (\nabla \times {\varvec{A}}) \mathrm {d}x + \int _{\Omega } {\varvec{A}}' \cdot \nabla \varphi \mathrm {d}x= & {} \int _{\Omega } {\varvec{A}}' \cdot {\varvec{\omega }}\mathrm {d}x \;\;\;\; \forall {\varvec{A}}' \in H_0(\mathrm {curl},\Omega ) \nonumber \\ \int _{\Omega } {\varvec{A}} \cdot \nabla \psi \mathrm {d}x = & {} 0 \quad \forall \psi \in H_0^1(\Omega ) \, . \end{aligned}$$

In contrast to to the Lagrange constraint, mass regularization proceeds as follows: given a small $\epsilon _\mathrm {reg}>0$, find ${\varvec{A}}^\epsilon \in H_0(\mathrm {curl},\Omega )$ such that

32 $$\begin{aligned} B^\epsilon ({\varvec{A}}',{\varvec{A}}^\epsilon ) = \int _{\Omega } (\nabla \times {\varvec{A}}') \cdot (\nabla \times {\varvec{A}}^\epsilon ) \mathrm {d}x + \int _{\Omega } \epsilon _\mathrm {reg} {\varvec{A}}' \cdot {\varvec{A}}^\epsilon \mathrm {d}x = \int _{\Omega } {\varvec{A}}' \cdot {\varvec{\omega }}\mathrm {d}x \, , \end{aligned}$$

holds for all ${\varvec{A}}^\prime \in H_0(\mathrm {curl},\Omega )$. To see that ${\varvec{A}}^\epsilon$ converges to ${\varvec{A}}$ as $\epsilon _{reg} \rightarrow 0$, we note that by (31), ${\varvec{A}}$ is divergence-free. Since the vorticity ${\varvec{\omega }}= \nabla \times {\varvec{u}}$ (cf. (23)) is divergence-free, testing (32) with gradient fields shows that ${\varvec{A}}^\epsilon$ is also divergence-free. The Friedrichs inequality (see, e.g., [17, Cor. 3.51]) then gives

33 $$\begin{aligned} c||{\varvec{A}}^\epsilon - {\varvec{A}}||^2_{L_2}\leqslant ||\nabla \times ({\varvec{A}}^\epsilon - {\varvec{A}})||^2_{L_2} \end{aligned}$$

34 $$\begin{aligned}\leqslant B^\epsilon ({\varvec{A}}^\epsilon - {\varvec{A}},{\varvec{A}}^\epsilon - {\varvec{A}}) = \epsilon _\mathrm {reg}({\varvec{A}},{\varvec{A}}^\epsilon - {\varvec{A}}) \, . \end{aligned}$$

The Cauchy–Schwarz inequality states the information about the upper limit of the error

35 $$\begin{aligned} \epsilon _\mathrm {reg}({\varvec{A}},{\varvec{A}}^\epsilon - {\varvec{A}}) \leqslant \epsilon _\mathrm {reg}||{\varvec{A}}||_{L_2}||{\varvec{A}}^\epsilon - {\varvec{A}}||_{L_2} \,. \end{aligned}$$

We observe that ${\varvec{A}}^\epsilon \rightarrow {\varvec{A}}$ converges as $\epsilon _\mathrm {reg} \rightarrow 0$

36 $$\begin{aligned} c||{\varvec{A}}^\epsilon - {\varvec{A}}||_{L_2} \leqslant \epsilon _\mathrm {reg}||{\varvec{A}}||_{L_2} \, . \end{aligned}$$

## Finite element formulation

**Fig. 3:**
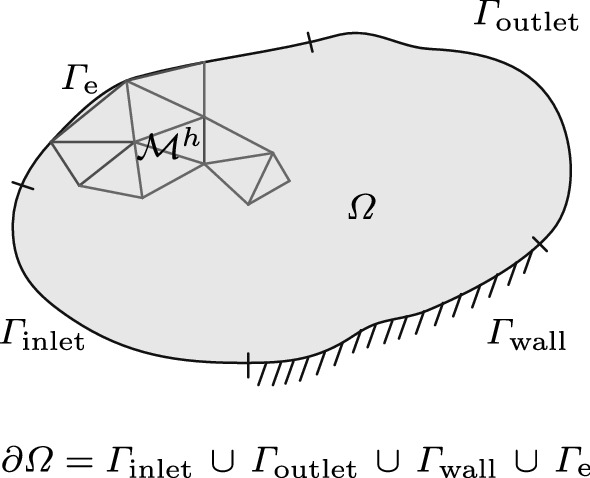
Schematic and definition of the decomposition domain $\Omega$, the triangulation $\mathcal {M}^h$, the boundary of the domain $\partial \Omega$ that consists of the boundary sections: wall $\Gamma _\mathrm{wall}$, inlet $\Gamma _\mathrm{inlet}$, outlet $\Gamma _\mathrm{outlet}$, and essential boundary $\Gamma _\mathrm{e}$

Two further steps are necessary to arrive at the finite element formulation of (32). At first, the continuous geometrical domain must be discretized by a mesh triangulation $\mathcal {M}^h \subset \Omega$ (see Fig. 3).

Secondly,an approximate finite element solution ${\varvec{A}}^h$ (Nédélec’s $H(\mathrm {curl})$ elements [38])

37 $$\begin{aligned} {\varvec{A}} \approx {\varvec{A}}^h = \sum _{e=1}^{m_e} {\varvec{N}}_e A_e \,\,\,\,\,\,\,\,\,\text {with}\,\,\,\,\,\, {\varvec{N}}_e \in V^h \, , \end{aligned}$$

is from a discrete function space $V^h \subset V$ and the test functions ${\varvec{(}}A')^h$ is chosen from the discrete function space $W^h \subset W$. Here, *e* denotes the degrees of freedom, $m_e$ the number of degrees of freedom in the triangulation, and $A_e$ the coefficients of the respective ansatz function. For lowest order edge elements of the first kind, the number of edges in the mesh is equivalent to the number of degrees of freedom, which are defined as the edge moments

38 $$\begin{aligned} A_e =\int _e {\varvec{A}}^h \cdot \mathrm {d}{\varvec{s}} \, , \end{aligned}$$

with ${\varvec{s}}$ as the tangential vector of edge *e*. Inserting the discretization into (32) for the unknown ${\varvec{A}}$ (the expression ${\varvec{A}}^\epsilon$ is omitted in the following, due to readability) as well as for the test function ${\varvec{A}}'$ results in the discrete form of the weak formulation

39 $$\begin{aligned}&\sum _a \sum _b \int _{\Omega } \Big ( ( A_a A_b (\nabla \times {\varvec{N}}_a) \cdot (\nabla \times {\varvec{N}}_b ) + \epsilon _\mathrm {reg} A_a A_b {\varvec{N}}_a \cdot {\varvec{N}}_b \Big ) \mathrm {d}x \nonumber \\&=\sum _a \left( A_a \int _{\Gamma _\mathrm{IO}} {\varvec{N}}_a \cdot ({\varvec{u}} \times {\varvec{n}}) \mathrm {d}s - A_a \int _{\Omega } {\varvec{N}}_a \cdot {\varvec{\omega }}\mathrm {d}x\right) \, , \end{aligned}$$

where $\Gamma _\mathrm{IO} = \Gamma _\mathrm{inlet} \cup \Gamma _\mathrm{outlet}$ denotes the boundary, where an inhomogeneous Neumann condition is applied. To sum up, the algebraic system of Eq. (39) is equivalent to

40 $$\begin{aligned} {\varvec{K}} \cdot \tilde{{\varvec{A}}} = {\varvec{f}} \, \end{aligned}$$

which is solved for the global vector of unknowns $\tilde{{\varvec{A}}}$. Each row in the global system matrix is associated with one specific test function unknown $A_a$. How to arrive at this standard matrix form can be found e.g. in [17, 53]. However, special care has to be taken when computing the inhomogeneous Neumann boundary in (39).

### Boundary term—transformation

The treatment of the boundary term in (39) is more complicated and involves the mapping to the integration space (integration coordinates ${\varvec{\xi }}= [\xi ,\eta ,\zeta ]^T$) and finite element specific operations to obtain the boundary shape functions

41 $$\begin{aligned} \int _{\Gamma _\mathrm{IO}} {\varvec{A}}' \cdot ({\varvec{u}}^{*}_\mathrm{v} \times {\varvec{n}}) \mathrm {d}s \, . \end{aligned}$$

Vector space transformations [17, 52] to the integration space involve a covariant transformation of the surface component and a transformation of the integral

42 $$\begin{aligned} \int _{\Gamma _\mathrm{ref}} {\varvec{A}}' \cdot \mathbb {J}^{-T} ({\varvec{u}}^{*}_\mathrm{v} \times {\varvec{n}}) \mathrm {det}\mathbb {J} \mathrm {d\xi d\eta } \, . \end{aligned}$$

At first, the normal on the surface ${\varvec{n}}$ points outside the connected base volume element. Then, an integration surface element transforms from the “physical” space ${\varvec{x}} = [x,y,z]^T$ to the “integration” space ${\varvec{\xi }}= [\xi ,\eta ,\zeta ]^T$ by

43 $$\begin{aligned} \mathrm {d}s = ||\frac{\partial {\varvec{x}}}{\partial \xi } \times \frac{\partial {\varvec{x}}}{\partial \eta }||_2 \mathrm {d\xi d\eta } \end{aligned}$$

that includes the definition of determinant in terms of the cross product. Thirdly, the Piola transformation projects the velocity $({\varvec{u}}^{*,\mathrm ic} \times {\varvec{n}})$ to the parameter space ${\varvec{\xi }}$ by the Jacobian $\mathbb {J}$

44 $$\begin{aligned} \mathbb {J} =\left( \frac{\partial {\varvec{x}}}{\partial \xi } , \frac{\partial {\varvec{x}}}{\partial \eta } \right) \, . \end{aligned}$$

Fourthly, the pseudo inverse of the Jacobian requires the inverse of the symmetric metric tensor $\mathbb {g}$^1^

45 $$\begin{aligned} \mathbb {g} = \mathbb {J}^T \mathbb {J} \, . \end{aligned}$$

Inserting these four ingredients into the inhomogeneous Neumann boundary yields

46 $$\begin{aligned} \int _{\Gamma _\mathrm{ref}} {\varvec{A}}' \cdot \mathbb {g}^{-T} \mathbb {J}^{T} ({\varvec{u}}^{*}_\mathrm{v} \times {\varvec{n}}) ||\frac{\partial {\varvec{x}}}{\partial \xi } \times \frac{\partial {\varvec{x}}}{\partial \eta }||_2 \mathrm {d\xi d\eta } \, . \end{aligned}$$

### Verification of boundary treatment

The verification example [48, 49], “cylinder in a crossflow”, demonstrates that the boundary condition with the choice $\beta =1$ includes the harmonic part of the decomposition inside the vortical part. The hypothesis is that for low Mach numbers, the vortical part will converge to the incompressible CFD solution. Helmholtz’s decomposition does not compute fluid dynamics but separates the compressible and vortical effects. Hence, it is self-evident that this decomposition can reproduce an incompressible flow. This verification case intends to prove that the decomposition with exact boundaries recovers the incompressible CFD solution. Therefore, we project the compressible CFD results to the incompressible function space using Helmholtz’s decomposition.

*Ansys Fluent* (ANSYS, Southpointe, PA/USA) is used to solve the unsteady viscous flow. Compressible and incompressible flow simulations are performed. The mesh of the circular 2D domain consists of quadrilateral finite-volumes that resolve the boundary layer and the wake. The flow field was resolved, according to the discretization parameters of [49]. The chosen time-step resolves the vortices in the wake of the cylinder, ”Von Karman vortex street”, characterized by the Strouhal number. During the compressible simulation, the fluid is modeled as an ideal gas. At the cylinder’s wall, a no-slip and non-penetration condition is enforced. A uniform velocity inlet at $\Gamma _\mathrm{inlet}$ and a pressure outlet condition at $\Gamma _\mathrm{outlet}$ complete the setup. Afterward, the benchmark simulation (incompressible flow) and the compressible flow (the subject of the decomposition) were interpolated to the finite element mesh using radial basis function interpolation [50]. However, the decomposition could be done on the same grid.

Mathematically, the decomposition on a domain with holes excites the harmonic function space. This harmonic term is known as the potential flow solution in physics. In the case of this vector potential formulation, having inhomogeneous Neumann boundaries $\beta =1$, the potential flow solution is included in the vortical component.

**Table 1:** Geometric and flow parameters of the “cylinder in a crossflow”

Parameter	Value	Description
Re	200	Reynolds number
M	0.03	Mach number
$U_\infty$	$10\,\mathrm{m}/\mathrm{s}$	Free stream velocity
$D=2a$	$0.02\,\,{\mathrm{m}}$	Cylinder diameter
St	0.2	Strouhal number

**Fig. 4:**
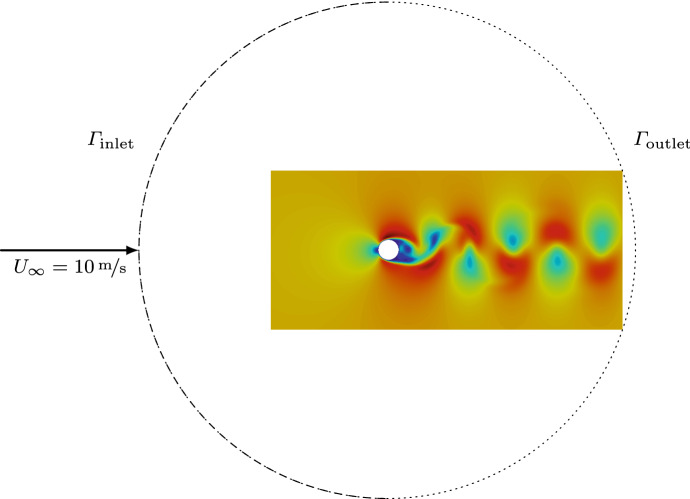
Schematic of the geometry and the flow configuration of the flow simulation with *Ansys Fluent*

In fluid dynamics, the simplest example of a homologically trivial domain is a cylinder, with a radius *a*, in a crossflow. Figure  4 and Table 1 summarize the geometrical and fluid dynamic properties. For the CFD simulation, we use a circular domain that surrounds the cylinder.

The potential flow theory provides an asymptotic approximation of the velocity potential $\Phi$ (see [19, 20])

47 $$\begin{aligned} \Phi = U_\infty \left( 1-\frac{a^2}{r^2} \right) r \sin \varphi . \end{aligned}$$

The “cylinder in a crossflow” involves no aeroacoustic feedback. Hence, the incompressible and the compressible flow converge as the Mach number decreases. A decomposition of the compressible velocity field, in its compressible component and vortical component, should recover the incompressible CFD solution.

**Fig. 5:**
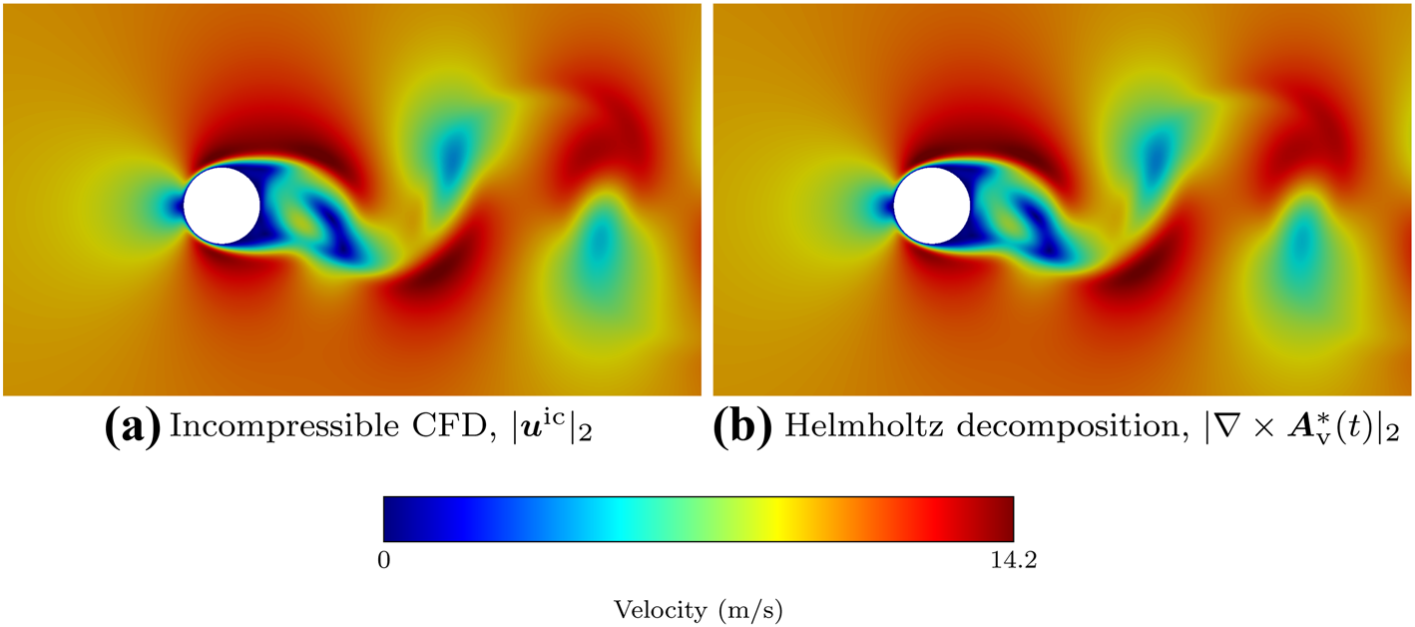
Comparison of the incompressible velocity (**a**) and the incompressible projection of a compressible velocity (**b**) [31]

Helmholtz’s decomposition computes the vortical component for a rectangular cutout of the CFD region. To extract the vortical part, we solve the curl-curl equation for the vector potential. The tangential velocity imposes the Neumann boundaries. Mass regularization guarantees the solvability of the Neumann problem. Figure 5 shows the similarity of the incompressible CFD and the incompressible (vortical and harmonic part) result of the Helmholtz decomposition. The velocity magnitude converges with a relative error of 0.6% between the two fields. The error is connected to a slight time lag of the CFD fields and due to the additional numerical procedures.

To conclude, we applied Helmholtz’s decomposition to a homologically trivial domain and proofed the hypothesis. Considering the vorticity distribution and the used Neumann boundaries, the simulation result contains the potential flow solution. Compared to the incompressible flow simulation, the inhomogeneous curl-curl equation provides accurate results.

## Application

Finally, we apply Helmholtz’s decomposition to a Mach 0.8 flow over a rectangular cavity [7].

**Fig. 6:**
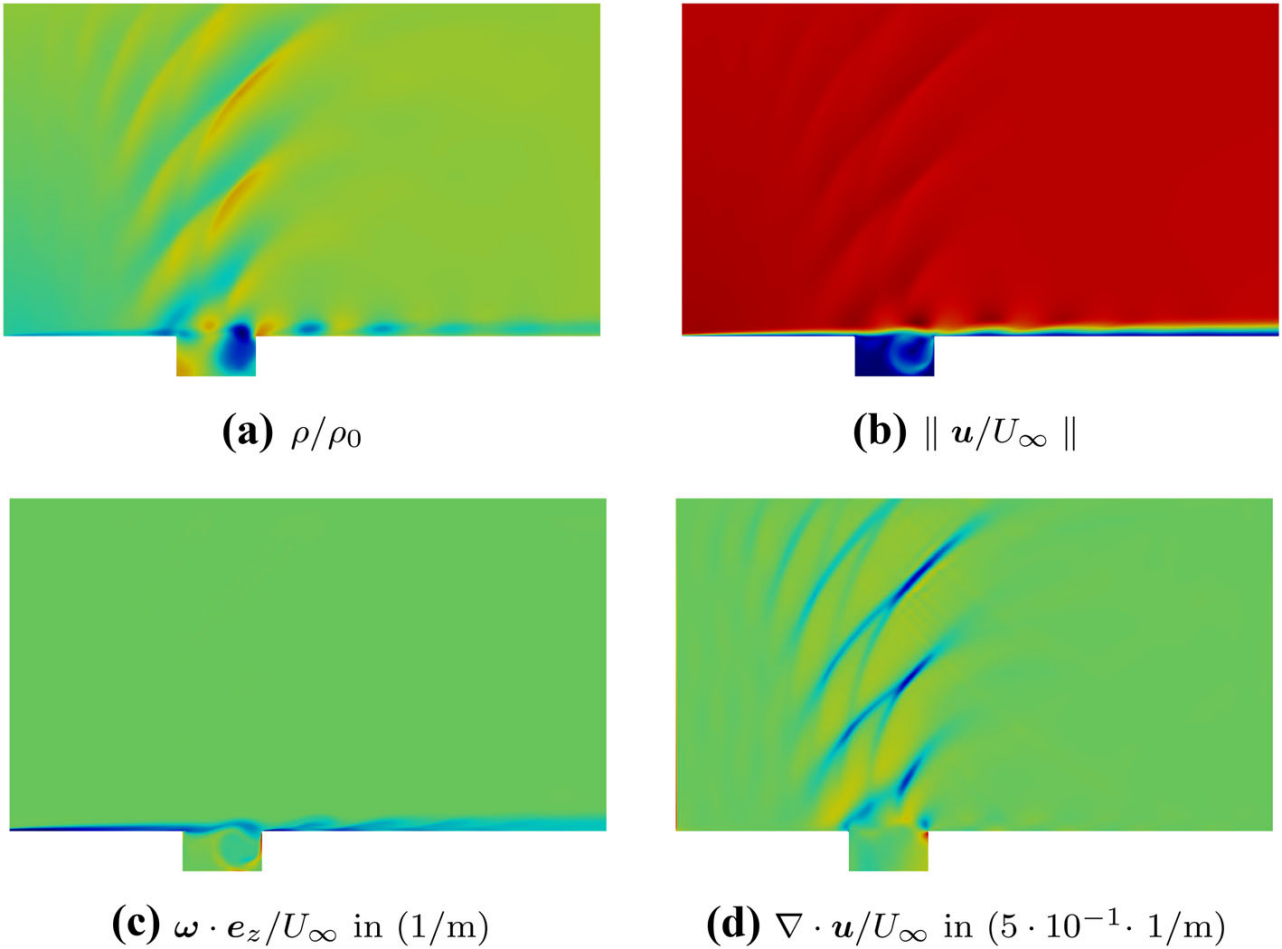
Plots of the flow results of the Mach 0.8 cavity. **a** The density displays compressible flow structures. **b** The magnitude of the flow velocity indicates a shear layer mode. **c** Vorticity field. **d** Rate of expansion [31]

Both vortical structures and acoustic waves are resolved in the direct sound simulation, a higher-order discontinuous Galerkin scheme in space and time [39]. The density field and the velocity field show a shear layer mode (see Fig.  6a, b). Density variations are present inside the cavity and convect downstream. Outside the cavity, propagating density variations indicate acoustic propagation. In the past, this result was used as post-processing of direct simulations of flow and acoustics [40–43]. Furthermore, the vorticity field (see Fig. 6c) and the rate of expansion (see Fig. 6d) are frequently used to distinguish between vortical structures and sound [7, 44, 45]. The acoustic waves originate at the cavity’s trailing edge. Typical cavity tone effects, like the Doppler’s shift and upstream amplification of the acoustic wave, are captured. This example shows the application of the inhomogeneous curl-curl problem.

**Fig. 7:**
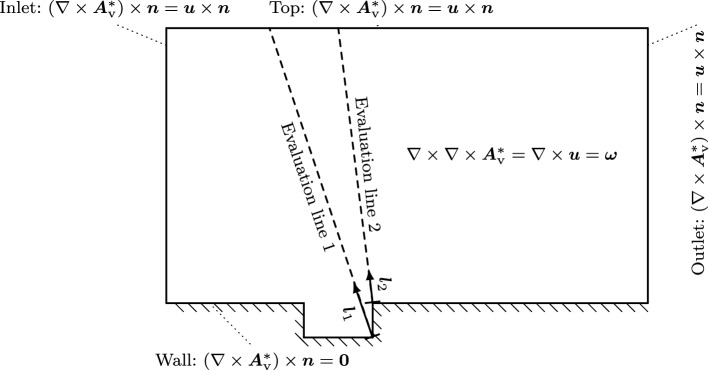
Schematic of the domain and the simulation setup of the vector potential formulation and the result evaluation lines

We solve (23) as an inhomogeneous mass-regularized Neumann problem forced by the vorticity (see Fig. 6c). Figure 7 illustrates the setup and boundary conditions. Firstly, the flow results on the discontinuous Galerkin mesh were interpolated to a refined quadrilateral finite element mesh [50]. The finite element mesh was found by a systematic mesh study that included four structured refinement stages. Secondly, the vorticity field was computed. Thirdly, the finite element simulation extracts the vortical velocity field.

At the inlet, top, and outlet boundary of the flow domain, an inhomogeneous Neumann boundary ($\beta =1$) is applied. At the wall, a homogeneous Neumann boundary models the no-slip condition.

**Fig. 8:**
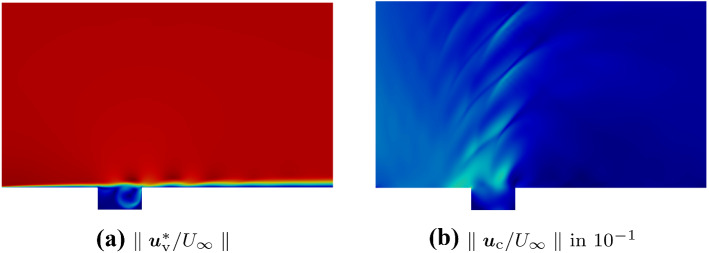
Plots of the decomposition of the Mach 0.8 cavity. **a** Vortical velocity component of the Helmholtz decomposition. **b** Compressible velocity component of the Helmholtz decomposition

Finally, the results of the computation are compared along the two evaluation lines as displayed in Fig. 7. We compare the results to the direct simulation of flow and acoustics [31]. Line ${\varvec{l}}_1$ includes the vortical flow inside the cavity, the shear layer, and the propagation of the sound waves. Since this line starts at a convex corner, we expect good agreement of the compressible part from the scalar-potential formulation [31] as well as the vector potential formulation. However, inside the shear layer differences will occur. Line ${\varvec{l}}_2$ includes the surrounding of the trailing edge and the second branch of the propagating sound waves. Again, we expect good agreement of the compressible part from the scalar-potential formulation [31] as well as the vector potential formulation with increasing distant to the wall.

Figure 8 shows the results of the decomposition. The Helmholtz decomposition is capable of separating the compressible effects (see Fig. 8b) from the vortical component (see Fig. 8a).

**Fig. 9:**
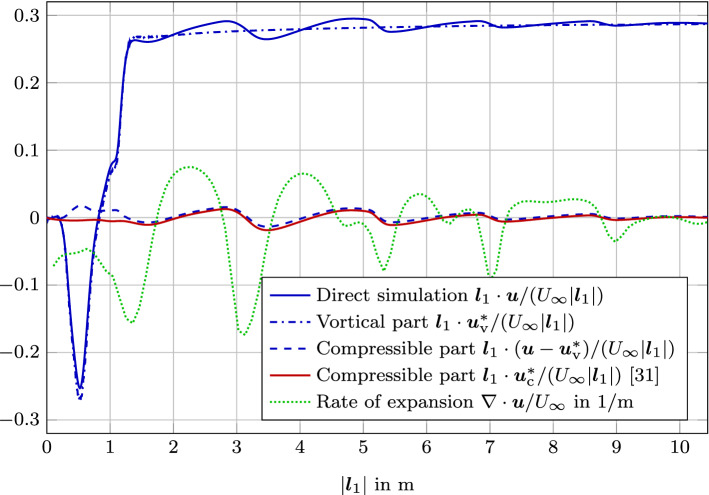
Comparison of the velocities projected onto the evaluation line ${\varvec{l}}_1$. Note that each compressible velocity part results from different equation solved: ${\varvec{u}} - {\varvec{u}}^*_\mathrm {v}$ is computed by (23) and ${\varvec{u}}^*_\mathrm {c}$ is computed by (20)

**Fig. 10:**
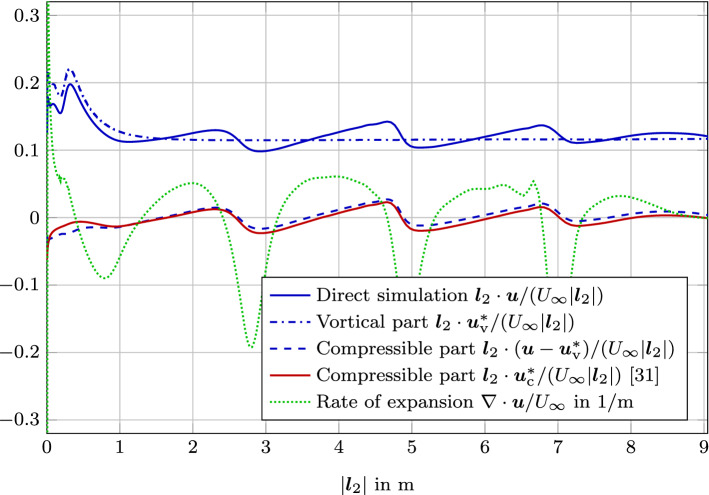
Comparison of the velocities projected onto the evaluation line ${\varvec{l}}_2$. Note that each compressible velocity part results from different equation solved: ${\varvec{u}} - {\varvec{u}}^*_\mathrm {v}$ is computed by (23) and ${\varvec{u}}^*_\mathrm {c}$ is computed by (20)

Now, the vortical component does not contain radiation patterns of acoustic waves, but all the vortical structures due to the shear layer and the edge interaction are still present.

**Fig. 11:**
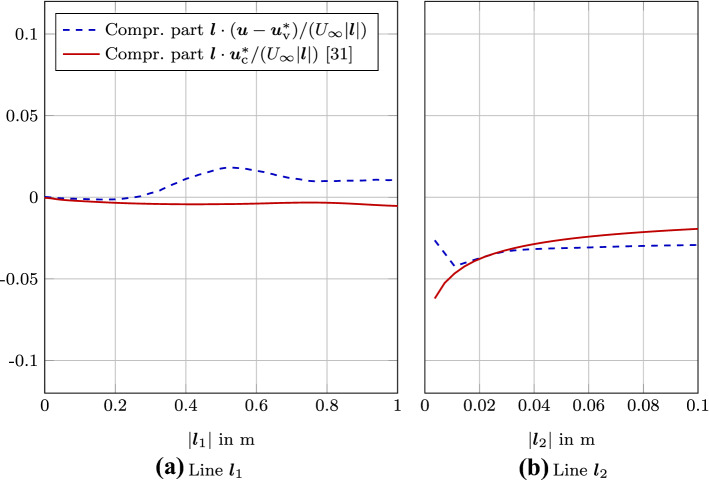
Comparison of the compressible velocities (${\varvec{u}} - {\varvec{u}}^*_\mathrm {v})$ computed by (23) and ${\varvec{u}}^*_\mathrm {c}$ computed by (20)) projected onto the evaluation line ${\varvec{l}}_1$ and ${\varvec{l}}_2$

When comparing the results of the decomposition along line ${\varvec{l}}_1$, we see that the direct simulation of flow and acoustics includes vortical effects and sound propagation (Fig. 9). After computing the vortical part ${\varvec{u}}^*_\mathrm {v}$, no sound propagation is visible in the results and the computed compressible part ${\varvec{u}}_\mathrm {c} = {\varvec{u}} - {\varvec{u}}^*_\mathrm {v}$ shows the characteristic sound radiation. At $|{\varvec{l}}_1|$ around one, the compressible part ${\varvec{u}}_\mathrm {c}$ deviates from the compressible velocity results ${\varvec{u}}^*_\mathrm {c}$ using (20). Fig. 11 shows the details of this comparison. The compressible velocity component ${\varvec{u}}_\mathrm {c} = {\varvec{u}} - {\varvec{u}}^*_\mathrm {v}$ solving Eq.  (23) has wavy components inside the cavity. In contrast to that, the ${\varvec{u}}^*_\mathrm {c}$ is dominated by the effects of the Neumann boundary condition at the wall. Comparing this deviation to the rate of expansion $\nabla \cdot {\varvec{u}}$, let us conclude that the ${\varvec{u}}_\mathrm {c} = {\varvec{u}} - {\varvec{u}}^*_\mathrm {v}$ is more accurate inside the cavity (see Fig. 11).

For evaluation line ${\varvec{l}}_2$, similar trends are visible (Fig. 10). As pointed out previously, the two different results of the compressible part deviate at the wall boundary. Note that each compressible velocity part results from different equations solved: ${\varvec{u}} - {\varvec{u}}^*_\mathrm {v}$ is computed by (23) and ${\varvec{u}}^*_\mathrm {c}$ is computed by (20). This is attributed to the singularity occurring for the scalar-potential formulation at re-entrant corners [31]. The deviation at the re-entrant corner at the starting point of line ${\varvec{l}}_2$ is enlarged in Fig. 11. The compressible velocity part ${\varvec{u}}_\mathrm {c} = {\varvec{u}} - {\varvec{u}}^*_\mathrm {v}$ is gradually approaching zero due to the no slip boundary condition. In contrast to that for the re-entrant the compressible part ${\varvec{u}}^*_\mathrm {c}$ (the result of (20)) is heading towards an non-physical singular point at the re-entrant corner. Furthermore, both methods can capture the sound propagation for $|{\varvec{l}}_2|$ values between two and nine.

These promising results support further investigation of Helmholtz’s decomposition as a tool to compute out of a compressible flow field the base flow and based on it, physically correct acoustic source terms.

## Conclusions

Within this contribution, we have demonstrated a physical and mathematical correct application of Helmholtz’s decomposition to compressible flow fields on finite domains. By including the harmonic part into the vortical part and exploring the orthogonality condition of the decomposed vector fields, the appropriate boundary condition for the curl-curl equation is derived and verified by the “cylinder in a crossflow” at low Mach number. Thereby the vortical part obtained by the decomposition converges to the incompressible flow solution. Besides, we applied Helmholtz’s decomposition to a Mach 0.8 flow over a rectangular cavity. The hypothesis that we can separate acoustic structures from vortical structures was demonstrated. The vortical component contains all the vortical structures generated by the shear layer and the edge interaction. Therefore, the derived method allows us to extract the pure vortical part from a compressible flow field on a restricted domain (non-zero flow at the boundaries). This so-called non-radiating base flow is the physically correct field from which acoustic source terms are obtained within computational aeroacoustics.

## Footnotes

1 The pseudo inverse represents the transformation property described by Monk [17] (Page 216).
